# Seismic Facies Analysis Using the Multiattribute SOM-K-Means Clustering

**DOI:** 10.1155/2022/1688233

**Published:** 2022-10-10

**Authors:** Zhaolin Zhu, Xin Chen, Haoran Ren, Liurong Tao, Jinsheng Jiang, Tong Wang, Mingxin Cheng, Shuaimin Ding, Rui Du

**Affiliations:** ^1^Hainan Institute of Zhejiang University, Yongyou Industrial Park, 11# Building, Yazhou Bay Science and Technology City, Yazhou District, Sanya 572025, Hainan, China; ^2^School of Geosciences, China University of Petroleum (East China), 66# Changjiang Xi Road, Huangdao District, Qingdao 266580, Shandong, China; ^3^Zhejiang University, 154# Yugu Road, Xihu District, Hangzhou 310027, Zhejiang, China

## Abstract

An accurate seismic facies analysis (SFA) can provide insight into the subsurface sedimentary facies and has guiding significance for geological exploration. Many machine learning algorithms, including unsupervised, supervised, and deep learning algorithms, have been developed successfully for SFA over the past decades. However, SFA and facies classification are still challenging tasks due to the complex characteristics of geological and seismic data. A multiattribute SOM-K-means clustering algorithm, which implements a two-stage clustering by using multiple geological attributes, is proposed and applied for SFA. The proposed algorithm can effectively extract complementary features from the multiple attribute volumes and comprehensively use the different attributes to improve the recognition ability of seismic facies. Experimental results show that the proposed algorithm improves clustering accuracy and can be used as an effective and powerful tool for SFA.

## 1. Introduction

The seismic facies analysis (SFA) is an important step in investigating the sedimentary spatial distribution in geological exploration and emphasizes the integration of seismic data into geology [1]. The traditional SFA algorithms rely on researchers' theoretical knowledge and experiences, which have the problems of low efficiency and personal subjectivity. Machine learning (ML) algorithms can help address the problems by performing SFA in a rigorous and repeatable way [2]. Although supervised machine learning (ML) methods, such as support vector machines (SVM), random forests (AF), and the supervised artificial neural network (ANN) are more attractive to the SFA in recent years, they require prior knowledge and more effort in labeling the interpreted geological datasets. The unsupervised ML methods, which include the Gaussian mixture model (GMM), self-organizing maps (SOM), *K*-means clustering and its updated versions, etc., are easier for SFA because they depend on the known geological information. The SFA algorithms based on GMM transform the seismic attributes into the parameter estimation problem of the Gaussian mixture model [3]. SOM can divide seismic facies by calculating waveforms and analyzing changes in seismic data [4–6]. *K*-means clustering is one of the classical pattern recognition algorithms, and its updated versions involve selecting multiple attributes representing different characteristics for SFA [7–10].

The *K*-means clustering algorithm [11] based on dividing is one of the simplest unsupervised machine learning algorithms, which can work well if clusters are separable but need to give the number of clusters in advance. Also, the clustering results are greatly affected by the initial cluster centers [12–14]. Yuan and Yang [13] analyzed four algorithms to solve the problem that K-values for the K-means algorithm needs to be set in advance and discussed the advantages and disadvantages of the four methods in the experimental results. Ahmed et al. [15] analyzed the problems of the *K*-means algorithm, such as the issues of initialization, inability to handle data with mixed types of features, and introduced the relevant research to solve the problems. SOM is an unsupervised network proposed by Kohonen [16], which has been widely used in many fields of data clustering [17–19]. SOM has a strong learning ability but cannot provide precise clustering results, and the convergence speed is slow [14, 20]. Miljković [21] introduced the SOM algorithm in detail, including its basic tenets, motivation, architecture, and applications, which is helpful for us to understand and master the SOM algorithm. Sahoo and Jha [22] proposed a novel hybrid self-organizing map (SOM) based ANN (SOM-ANN-GA) method for the prediction of lithology, and the model can achieve the most rational prediction of lithology patterns. Cottrell et al. [23] present the state-of-art on the theoretical aspects of SOM, shows how to extend original SOM to non-numerical data, and finally provide some directions to go further. In this paper, we propose a multiattribute SOM-*K*-means clustering algorithm, which implements a two-stage clustering by using multiple geological attributes, and apply it for SFA. In the proposed algorithm, SOM aims at attaining the cluster number and centers of multiattributes data, and the following K-means clustering algorithm improves the clustering feature accuracy.

The rest of this paper is organized as follows.  Section 2 outlines the related work on the basic K-means clustering and SOM networks.  Section 3 presents the framework of our SOM-based K-means clustering algorithms. In Section 4, the experiments are implemented, and the conclusion is given in Section 5.

## 2. Related Work

### 2.1. Overview on K-Means Clustering Algorithm

The *K*-means clustering algorithm is a kind of centroid-based clustering algorithm, which randomly chooses initial cluster centers and then assigns each point in the cluster closest to the cluster center [24, 25]. Given the dataset **X**={**x**_*i*_}_*i*=1_^*N*^ with *N* observations, where **x**_*i*_ ∈ *ℝ*^*D*^, which is grouped into *K* homogeneous clusters with the centroids **C**={**c**_*j*_}_*j*=1_^*K*^ ∈ *ℝ*^*K*×*D*^. The algorithm uses Euclidean distance to find the distance between data points and cluster centers. The objective function is given by the following equation:

(1) $$\begin{array}{c} d=\overset{K}{\underset{j=1}{\sum }}\overset{N}{\underset{i=1}{\sum }}{\left\|\left(x_{i}-c_{j}\right)\right\|}^{2}\text{,} \end{array}$$

where **c**_*j*_ represents the jth cluster center, and the **x**_*i*_ represents the ith sample vector in the dataset **X**, and the sample vector contains one or more seismic attributes. With each iteration of the algorithm, cluster centers change, and the target of the *K*-means clustering algorithm is to minimize the distance *d*.

The main steps of a standard K-means clustering algorithm include the following [26, 27]:

1. Preset the cluster number of samples and randomly select *k* points in the sample point set as the initial cluster centers.
2. Calculate the distances from sample points to each cluster center, and the points are grouped into the corresponding clusters according to the principle of the nearest distance.
3. In the new clusters, new cluster centers are reselected and recalculated the Euclidean distance and reassigned each data point to each cluster.
4. Repeat steps (3) until the convergence criterion is met.

Through the steps of the *K*-means clustering algorithm, it can be found that the algorithm is easy to understand and operate. However, its weaknesses are also conspicuous. The cluster number in the algorithm is set in advance and will not change during the iteration process; if the selection of the cluster number is not good for clustering, it will directly affect the clustering results. Another is that the initial clustering centers of the algorithm are randomly selected, and the clustering results easily fall into the optimal local solution.

### 2.2. SOM Network

**Figure 1:**
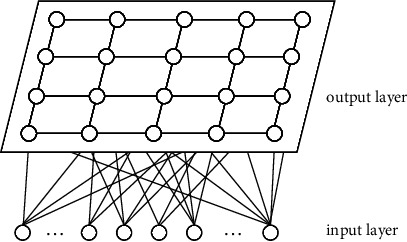
The SOM network structure.

The SOM is a prominent unsupervised neural network [28], which consists of the input layer and output layer (or competition layer); the typical structure of a SOM network is shown in Figure 1 [29, 30]. The function of the input layer is mainly to receive information and transmit the input mode to the output layer. The number of neurons is generally the number of samples; the function of the output layer is for comparative analysis of input and output and the classification of patterns; the number of neurons is usually the number of clusters [14].

The steps of a SOM network can be summarized as follows:

(1) Initialize the weight of each of all the neurons in the output layer to a small random number.
(2) A sample vector **X**={**x**_*i*_}_*i*=1_^*N*^ is randomly selected from the dataset, and calculate the distance between the sample vector and neurons. Euclidean distance is usually used for the calculation, the equation is as follows:
  (2) $$\begin{array}{c} d_{j}=\sqrt{\overset{N}{\underset{i=1}{\sum }}{\left(x_{i}-w_{ji}\right)}^{2}}\text{,} \end{array}$$
where the **x**_*i*_ represents the *i*th sample vector in the dataset *X*, the **w**_*ji*_ represents the weights vector that joints input node and the jth output node.
(3) Select the neuron *b* with the smallest distance as the best matching unit (BMU):
  (3) $$\begin{array}{c} \left\|x-w_{b}\right\|=\min\left\|x-w_{j}\right\|\text{,} \end{array}$$
where ‖·‖ represent the Euclidean distance, **w**_*b*_ is the closest vector to **x** on the map.
(4) Find the neighbor neurons according to the predefined neighborhood function, and then update its weight. The weight is updated according to the following equation:
  (4) $$\begin{array}{c} w_{j}^{t+1}=w_{j}^{t}+\left[\alpha^{t}h\left(\left\|r_{BMU}-r_{i}\right\|,t\right)\right]*\left(x^{t}-w_{j}^{t}\right)\text{,} \end{array}$$
where **x**^*t*^ is the input sample of iteration *t*, **w**^*t*^, and **w**^*t*+1^ represent the weights before and after the update respectively, *α*^*t*^ is the learning rate, *h* is the neighbor kernel of the best matching unit.
(5) Complete a round of iterations and return to step 2 until the set number of iterations is met.

The SOM network has the advantages of strong explanatory and learning ability, visualization, and so on. However, its convergence speed is slow, and the clustering accuracy is poor for the nonlarge volume of samples. So, it is not suitable for SFA.

## 3. SOM-K-Means Clustering for SFA

**Figure 2:**
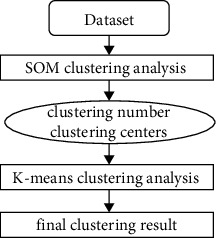
Flow chart of the SOM-K-means algorithm.

Considering the specialties of SOM networks and the *K*-means clustering, we combine them to propose the SOM-*K*-means algorithm. The proposed algorithm is divided into two steps. Firstly, the SOM network is used to train the seismic dataset in a short amount of time. The number *k* of clusters is determined according to the winning situation of neurons in the output layer, and the weight of neurons is taken as the initial cluster centers. During the second step, the *K*-means clustering algorithm is used to refine the clustering results of SOM and improve the clustering accuracy. The flow chart of the proposed SOM-*K*-means algorithm can be described in Figure 2.

**Figure 3:**
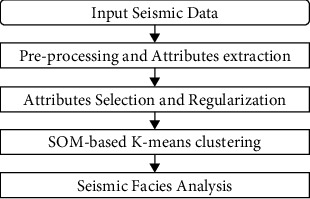
SFA process based on the multiattribute SOM-K-means algorithm.

The proposed SOM-*K*-means algorithm is implemented by using the multiple seismic attributes for SFA, which can not only effectively use the information of each seismic attribute but also realize the complementarity between attributes. The algorithm generally consists of the following steps: seismic data input, preprocessing and attribute extraction, attribute selection and regularization, SOM-based K-means clustering, and seismic facies identification (Figure 3).

The input seismic data needs several steps before clustering. Preprocessing is usually implemented by filtering. The extracted attributes represent the different features of seismic facies, so the right attributes are selected as the input components for our objectives. During this process, we need to consider the correlation between attributes. The higher the correlation coefficient of different seismic attributes, the more geological information represents the similarity between attributes. In optimizing seismic attributes based on the comprehensive effect of seismic attributes, try to select attributes with low correlation coefficients to avoid repeated interference from attributes. However, the magnitude among attributes is different because they are calculated with the different formulas. The normalization step is essential, and the normalized processing algorithm is used to limit the seismic data to 0∼1. Also, the SOM-*K*-means clustering is implemented to analyze the seismic attributes. Finally, the seismic phase identification results are outputted. The proposed iterative SOM-*K*-means clustering is the critical step of the above SFA process, and the improved clustering algorithm can not only make up for the shortcomings of the two algorithms but also improve the clustering accuracy.

## 4. Seismic Facies Analysis Experiments

### 4.1. Seismic Facies Analysis for the Sandstone Data

**Figure 4:**
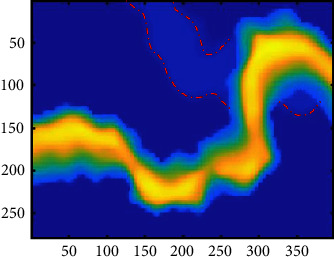
A schematic slice of the sand slice. The slice contains two sand bodies, and the sand body characteristics shown in the red box are weak.

**Figure 5:**
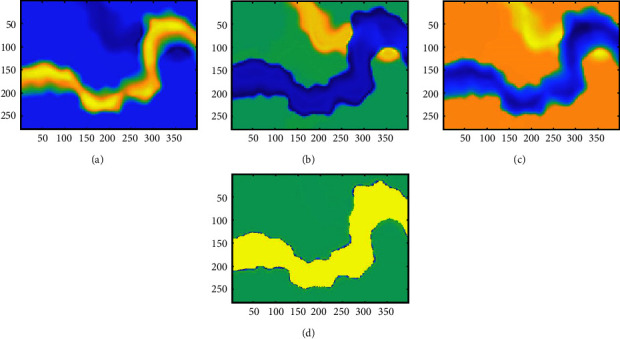
Seismic attributes of the sand body slice. (a) Instantaneous amplitude. (b) Instantaneous frequency. (c) Instantaneous phase. (d) Event.

**Figure 6:**
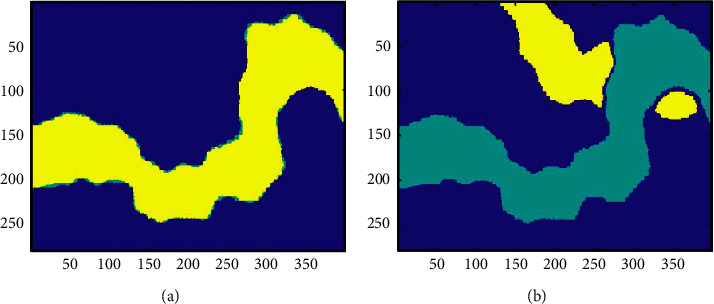
(a) The clustering result of event attribute. (b) The clustering result of multiple attributes. Both are the results of clustering analysis using the *K*-means algorithm, and the number of clusters set in advance is 3.

In this section, we implement SFA on the Sandstone data of a work area. A schematic slice of the Sandstone data can be seen in Figure 4. The slice contains two sand bodies, one of which has weak characteristics (red box) and is challenging to identify. By analyzing the sensitivity of seismic attributes to the reservoir, the instantaneous amplitude, the instantaneous frequency, the instantaneous phase, and event attributes (Figure 5) are extracted for cluster analysis. Due to the different extraction algorithms for different seismic attributes, the magnitude difference between the data is large, and the seismic attributes need to be normalized as said before. It can be seen from the attribute slice that the event attribute can only reflect the characteristics of one sand body; therefore, when clustering the event attributes with the *K*-means clustering algorithm, the sand body with weak characteristics cannot be identified (Figure 6(a)). To avoid the inaccurate identification of seismic facies caused by insufficient single-attribute information, we carry out multiattribute clustering analysis and comprehensively use the efficient information of different attributes. We also used the k-means algorithm to perform clustering analysis using multiple attributes (Figure 6(b)). As shown in Figure 6, under the same number of clusters, multiattribute clustering can effectively identify the characteristics of two sand bodies and ensure the reliability of seismic facies identification.

**Figure 7:**
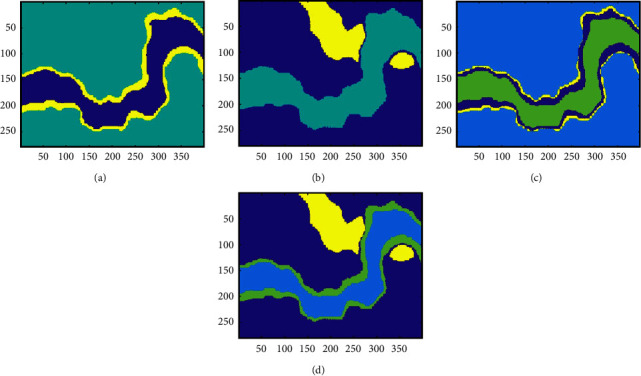
Comparison of the *K*-means clustering results with different cluster numbers. (a) and (b) show the clustering results of the *K*-means algorithm when *k* = 3. (c) and (d) show the clustering results of the *K*-means algorithm when *k* = 4.

*The K*-means clustering algorithm has two weaknesses: it needs to set the number of clusters in advance and randomly select the clustering center. We will analyze the two weaknesses, respectively. For real data, the number of clusters is generally difficult to define. The choice of clustering number will directly affect the final clustering results, as shown in Figure 7. Some studies test the number of clusters one by one and choose the best number for the *K*-means workflow. This way is undesirable, taking a lot of computing costs, and there are also human factors in selecting the best among different clustering results. Comparing the clustering results with the same number of clusters, it can be seen that the *K*-means clustering algorithm is sensitive to the initial clustering center and the clustering results change with the clustering center (Figure 7). At the same time, the *K*-means clustering algorithm needs to constantly adjust the sample classification and calculate the latest clustering center. Improper selection of the initial clustering centers increases the number of iterations and needs a more computation load.

**Figure 8:**
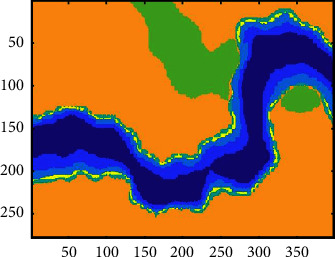
The clustering result of the SOM-*K*-means algorithm. The number of clusters is 7, which is obtained by the SOM algorithm.

Therefore, we use the SOM-*K*-means algorithm for clustering and the SOM network to provide the initial cluster number and cluster centers for the *K*-means clustering algorithm. The clustering result of the SOM-*K*-means algorithm is shown in Figure 8. The figure shows that the sand bodies with weak characteristics have been effectively identified, and the analysis of sand bodies with strong features is more detailed. The SOM-*K*-means algorithm effectively makes up for the weaknesses of the *K*-means clustering algorithm, improves the accuracy of clustering results, and is conducive to the accurate analysis of seismic facies.

### 4.2. Seismic Facies Analysis for F3 Data

**Figure 9:**
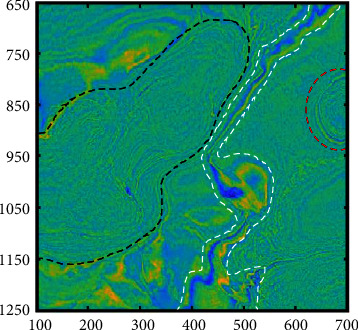
A horizontal slice of the North Sea F3 data at 1920 ms. The seismic phase of this slice is relatively complex, mainly including the gas chimney (red line), salt dome (black line), and river channel (white line).

**Figure 10:**
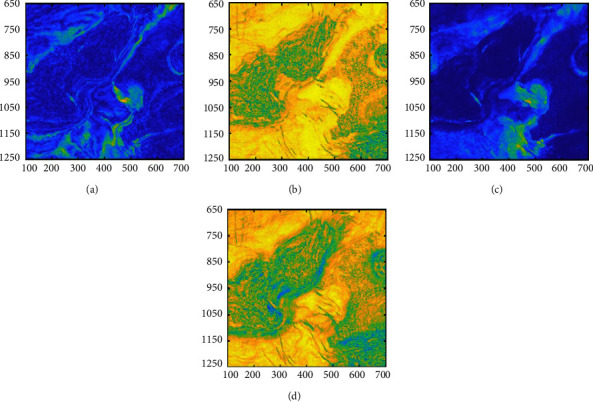
Seismic attributes of the horizontal slice (a) Instantaneous amplitude. (b) Dip. (c) Energy. (d) Similarity.

A 3D seismic data volume from the F3 block of the Dutch part of the North Sea is applied in this experiment, which is composed of 601 in-lines from 650 to 1250 and 601 cross-lines from 100 to 700, and the sampling rate is 4 ms. We select the horizontal slice at 1920 ms (Figure 9); the slice includes several phase characteristics such as the gas chimney (red line), salt dome (black line), and river channel (white line). By analyzing the sensitivity of seismic attributes to the reservoir, the instantaneous amplitude, dip, energy, and similarity attributes are selected as input views, and the four attributes are normalized. The visualization of the attributes is shown in Figure 10.

**Figure 11:**
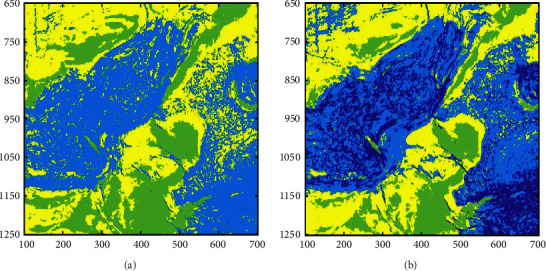
Multiattribute clustering results of the different algorithms. (a) Clustering results of the SOM network. (b) Clustering results of the SOM-K-means algorithm.

The SOM and the SOM-*K*-means algorithms comprehensively use four attributes for multiattribute clustering analysis; the number of clusters is 4, and the clustering results are shown in Figure 11. It can be seen from Figure 11(a) that the SOM network can classify the main sedimentary facies (salt hills, rivers, and gas chimneys) in the 1920 ms time slice. However, the algorithm can only depict the outline of sedimentary facies, and its ability to portray details is poor. The clustering result reflects the shortcomings of the SOM network, which cannot provide us with precise clustering results, and clustering accuracy for the nonlarge volume of samples is poor. Therefore, we introduce the SOM-K-means algorithm and use the K-means clustering algorithm to refine the clustering result of the SOM stage. As shown in Figure 11(b), the SOM-K-means algorithm result is visible and clear, which is conducive to the identification and analysis of seismic facies.

**Figure 12:**
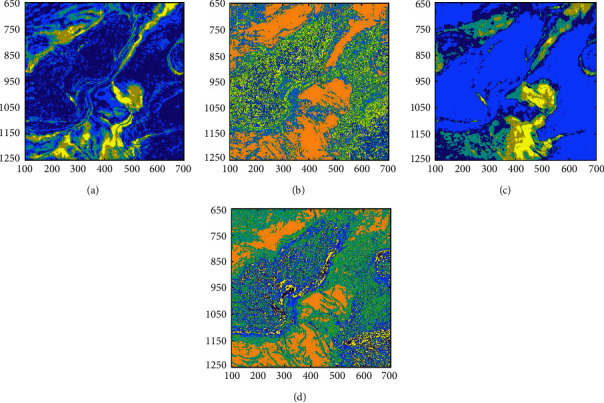
SOM-K-means algorithm clustering results for the different attributes (a) Instantaneous amplitude. (b) Dip. (c) Energy. (d) Similarity.

Figure 12 shows the following single attribute clustering results: (a) the single dip; (b) the single coherence; (c) the single instantaneous amplitude attribute; and (d) similarity. The instantaneous amplitude and energy highlight the river, and the recognition ability of the salt dome and the gas chimney is poor. Also, the dip and similarity results only highlight the salt dome and the gas chimney. Thus, multiattribute clustering can use the useful information of each attribute to cluster, which allows us to better understand seismic facies.

### 4.3. Seismic Facies Analysis for the Beach-Bar Sand Data

**Figure 13:**
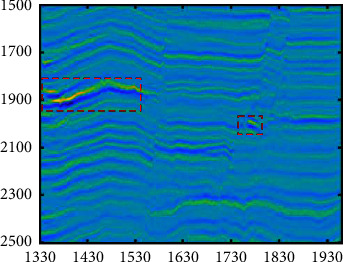
Seismic attributes of the seismic section. The profile contains seismic facies such as fault and bright spot reflection (in the red box in the figure).

This section tests the proposed algorithm by applying it to other 3D real beach-bar sand data with well-developed faults. The three-dimensional data volume consists of 631 in-lines from 1330 to 1960 and 301 cross-lines from 780 to 1080, and the sampling rate is 2 ms. For this analysis, we used a seismic section containing the fault and a time-interval window lying above fault zones, as shown in Figure 13.

**Figure 14:**
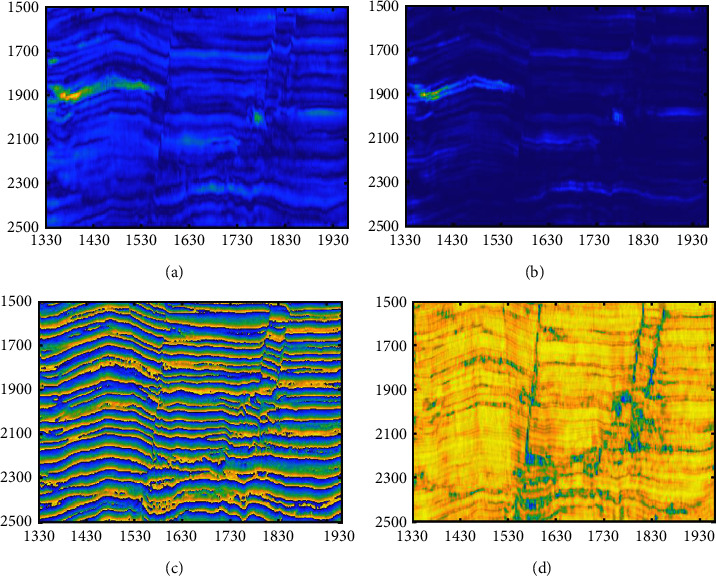
Seismic attributes of the seismic section (a) Instantaneous amplitude. (b) Energy. (c) Instantaneous phase. (d) Similarity.

We select the cross-line 300 section (Figure 13) with the in-lines from 1330 to 1960 and time from 1500 to 2500 ms, and seismic facies such as fault and bright spot reflection (within the red box in the figure) can be seen from the profile. By analyzing the sensitivity of seismic attributes to the reservoir, combined with the correlation between the attributes, the instantaneous amplitude, energy, instantaneous phase, and similarity properties of this section are extracted, and these four properties are normalized. The visualization of these three attributes is shown in Figure 14.

**Figure 15:**
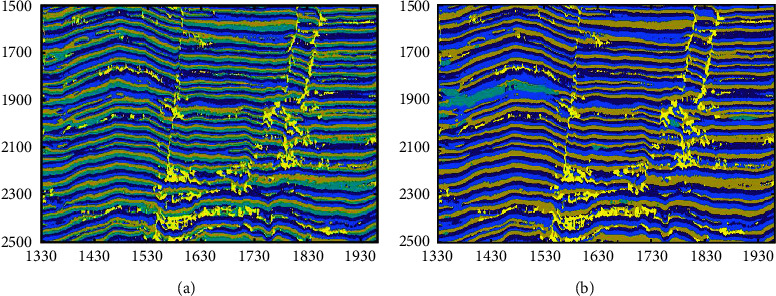
Multiattribute clustering results of the different algorithms. (a) Clustering results of the K-means algorithm. (b) Clustering results of the SOM-*K*-means algorithm.

We use the K-means and the SOM-*K*-means algorithms to cluster the profile, and the results are shown in Figure 15. It can be seen from the figures that the *K*-means clustering algorithm and the SOM-K-means algorithm can well identify faults and characterize the fault strike in the profile. The SOM-*K*-means algorithm is better for the description of bright spots than the *K*-means clustering algorithm. Two bright spot reflections in the seismic profile are clustered by the SOM-K-means algorithm (as shown in the red box in Figure 15(b)), while the *K*-means clustering algorithm cannot recognize bright spot reflections. Comparing the efficiency of the two algorithms (as shown in Table 1), because the SOM network provides the initial clustering center, the computing efficiency of the *K*-means clustering algorithm (the second stage of the SOM-*K*-means algorithm) has been greatly improved, and the clustering time of the SOM-*K*-means algorithm is saved by nearly 1/3. It can be seen that the SOM-*K*-means algorithm is more suitable for SFAs of seismic data.

**Figure 16:**
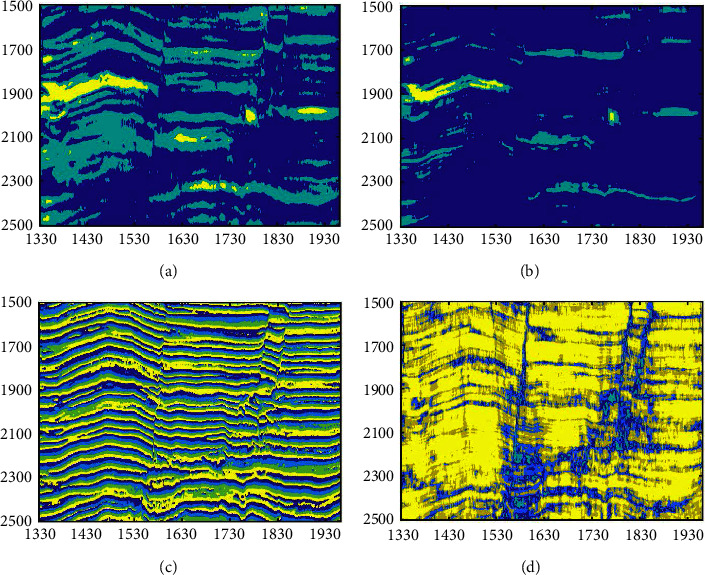
The SOM-K-means algorithm clustering results for the different attributes (a) Instantaneous amplitude. (b) Energy. (c) Instantaneous phase. (d) Similarity.

The multiattribute clustering compares with single-attribute clustering.  Figure 16 shows the results of the following: (a) the single instantaneous amplitude attribute; (b) the single energy; (c) the single instantaneous phase attribute; and (d) the single similarity. The instantaneous amplitude and energy result only highlight the bright spot reflection, the instantaneous phase and similarity highlight the faults, and the single attribute cannot cluster all three facies. Thus, multiattribute clustering is better for analyzing more facies simultaneously.

## 5. Conclusions

In this paper, we propose the SOM-*K*-means algorithm, which combines the SOM neural network and the *K*-means clustering algorithm for seismic facies analyses, and successfully apply it to the real data. The SOM-*K*-means algorithm not only improves the accuracy of SOM results but also solves the problems of the *K*-means clustering algorithm that the number of clusters needs to be given in advance and the initial cluster centers are to be defined randomly. Because the SOM network provides the good initial clustering centers for the *K*-means clustering algorithm, it can not only prevent the *K*-means clustering algorithm from falling into optimal local solutions but also improve the efficiency of the *K*-means clustering algorithm. In the results of the real seismic data clustering, we show the benefits of the SOM-*K*-means algorithm over using the SOM network or *K*-means clustering as stand-alone algorithms. A single seismic attribute can only represent part of the seismic facies information, which cannot meet the current requirement of seismic facies identification. The idea of multiattribute clustering considers the differences in data from different perspectives. It comprehensively utilizes the features of different attributes to make the division of seismic facies more accurate and reasonable. The application of the multiattribute SOM-K-means algorithm to real seismic data has achieved good results, which shows that the algorithm can provide an effective SFA.

## Figures and Tables

**Table 1:** Efficiency comparison between SOM and SOM-K-means clustering for SFA.

Algorithm	Iterations	Time
*K*-means	67	157s
SOM-*K*-means	39	105s

## Data Availability

The Sandstone and Beach-Bar Sand that support the findings of this study can be requested from the corresponding author upon request. F3 data used in the second test in this paper are open, which can be download from the website of dGB Earth Sciences.
